# Advantages of Photon-Counting Detector CT in Aortic Imaging

**DOI:** 10.3390/tomography10010001

**Published:** 2023-12-19

**Authors:** Chiara Zanon, Filippo Cademartiri, Alessandro Toniolo, Costanza Bini, Alberto Clemente, Elda Chiara Colacchio, Giulio Cabrelle, Florinda Mastro, Michele Antonello, Emilio Quaia, Alessia Pepe

**Affiliations:** 1 Department of Radiology, University of Padua, 35128 Padua, Italy; 2 Department of Radiology, Fondazione Toscana Gabriele Monasterio, 56124 Pisa, Italy; 3 Vascular and Endovascular Surgery Section, Department of Cardiac, Thoracic, Vascular Sciences and Public Health, University of Padua, 35128 Padua, Italy; 4 Division of Cardiac Surgery, University of Padua, 35128 Padua, Italy

**Keywords:** aortic imaging, vascular, photon-counting, CT angiography, dose exposure, contrast agents, EVAR, endoleaks

## Abstract

Photon-counting Computed Tomography (PCCT) is a promising imaging technique. Using detectors that count the number and energy of photons in multiple bins, PCCT offers several advantages over conventional CT, including a higher image quality, reduced contrast agent volume, radiation doses, and artifacts. Although PCCT is well established for cardiac imaging in assessing coronary artery disease, its application in aortic imaging remains limited. This review summarizes the available literature and provides an overview of the current use of PCCT for the diagnosis of aortic imaging, focusing mainly on endoleaks detection and characterization after endovascular aneurysm repair (EVAR), contrast dose volume, and radiation exposure reduction, particularly in patients with chronic kidney disease and in those requiring follow-up CT.

## 1. Introduction

Over the years, aortic imaging has evolved significantly with the development of new technologies, such as computed tomography (CT) and Photon-Counting CT (PCCT) [1]. Historically, angiography was the primary method for visualizing blood vessels, but the introduction of CT technology in the late 1970s marked a transformative shift due to its technological advantages. The subsequent development of ECG-triggered CT angiography (CTA) in the 1990s established a new standard for aortic imaging by reducing artifacts and enhancing precision [2,3]. Multidetector-row CT (MDCT) has further advanced vascular imaging, proving to be invaluable in diagnosing a range of vascular conditions, from acute aortic syndromes to vasculitis and emergencies such as gastrointestinal bleeding. It has also played a crucial role in preprocedural planning for interventions such as valve replacement [4]. The main indication for CTA in the evaluation of acute chest pain aims to exclude conditions of clinical emergency, such as obstructive coronary artery disease (CAD), aortic dissection [5], and pulmonary embolism [6]. To obtain quality reports, CT imaging requires approved acquisition protocols and post-processing image analysis using advanced software. Another limitation is exposure to ionizing radiation and the necessity of administering iodinated contrast media, which limits CT use in children, pregnant women, and patients with kidney disease.

Photon-counting CT (PCCT) provides high image quality and enhanced spatial resolution. Using detectors that count the number and energy of photons in multiple bins, PCCT offers several advantages over conventional CT, including higher spatial and contrast resolutions, fewer artifacts, and lower radiation doses [7]. Since its introduction in 2014, PCCT has become a well-established technique used in cardiac imaging to assess coronary artery disease (CAD) [1,7]. Rajagopal et al. demonstrated that PCCT exhibited superior accuracy in detecting plaque composition in coronary phantoms, compared to standard CT [8]. PCCT reconstructions showed the highest overall performance in the quantitative evaluation of lumen diameter because high-resolution images have a more precise rendition of contrast shape boundaries and are less susceptible to metal blooming artifacts. PCCT images were preferentially selected for qualitative evaluation [9]. Although high-resolution PCCT produces higher noise levels, it is less affected by artifacts and blooming caused by stents, leading to better quality images [9]. However, the application of PCCT in aortic imaging remains limited.

Recent studies have demonstrated the potential of PCCT in detecting and characterizing endoleaks (Els) after EVAR. PCCT shows endoleak detection capabilities comparable to those of traditional CT, reducing radiation exposure [10,11]. Bicolor K-edge imaging and dual-contrast agent protocols in PCCT allowed accurate characterization of ELs within the thoracic aorta. Furthermore, PCCT low-volume contrast protocols have shown promising results in reducing the contrast agent volume without compromising image quality [12]. This is particularly relevant for patients with chronic kidney disease or those requiring frequent follow-up imaging. Virtual monoenergetic images (VMI) reconstructed at optimal energy levels demonstrated improved contrast-to-noise ratios compared with energy-integrating detector (EID) CT, highlighting the potential of PCCT in achieving higher image quality while minimizing contrast-related risks [13].

PCCTs ability to enhance diagnostic accuracy, reduce the risks associated with contrast agents, and minimize radiation exposure makes it a valuable tool for the management of aortic conditions. As we continue to advance the integration of PCCT into clinical practice and explore its broader applications, this technology promises to reshape the landscape of aortic disease diagnosis benefiting patients with a wide range of conditions, particularly those undergoing EVAR, and those with renal issues. The future of vascular imaging appears brighter than ever, owing to these remarkable technological advancements.

This review begins with an overview of PCCT technology and its key benefits. The main discussion centers on the use of PCCT in aortic imaging applications, omitting small vessel, coronary, and carotid imaging considerations. A comprehensive review of the scientific literature was conducted in September 2023 via PubMed, employing search terms related to the aortic imaging by PCCT [“aorta OR aortic photon counting CT” or “photon counting CT endoleak”, “aorta photon counting CT OR contrast media volume reduction OR contrast media quantity reduction” and “aorta photon counting CT radiation dose”]. All articles concerning coronary imaging, head and neck vessel imaging, pulmonary vessels, small vessels, experimental animals, reviews, and those unrelated to photon counting CT were excluded. Moreover, we also excluded articles not written in English. Thus, we selected 10 studies: two prospective studies, four retrospective studies, one case report, and three phantom experimental studies.

This review aimed to synthesize the available literature and provide an overview of the current use of PCCT in aortic imaging (Table 1).

## 2. Photon-Counting Detector—Technical Considerations

The X-ray detector is a major component of a CT scanner and is critical for image formation and radiation dose. As X-rays leave the patient, they are picked up by the detectors and transmitted to a computer. The energy transported by radiation is converted into forms that can be visually or electronically recognized. Photons are absorbed by the detector and energy transfer occurs by ionization. The number of ionizations per photon is proportional to the energy of the absorbed photon and depends on the energy necessary to produce an electron pair in the detector [20].

A photon-counting detector (PCD) directly measures the energy of each photon and converts it into an electrical signal. PCD quantifies the number of photons and divides the X-ray energy spectrum into multiple bins. This technology offers several advantages over conventional CT, including improved spatial and contrast resolution, reduced image noise and artifacts, lower radiation exposure, and the ability to perform multienergy/multiparametric imaging based on the atomic properties of tissues. This enables the use of different contrast agents and enhances quantitative imaging [21]. PCCT also provides the capability to differentiate between materials based on the energy of incoming photons. Conventional CT uses energy-integrating detectors (EID) with scintillator elements that convert X-rays into visible light, which is then detected by a photodiode [18]. The photodiode indirectly measures the energy of the X-ray photons. Finally, the electrical signal is amplified and converted into a digital signal. The EID weighs the measured signal according to the energy of the detected photon; higher-energy photons generate stronger signals than lower-energy photons. In addition, the detector integrates the energy from all the detected photons without providing any information about the energy of the individual photons [22]. Instead, PCCT directly converts X-ray photons into an electrical signal by applying a high voltage to a semiconductor sensor between the cathode and the pixelated anode [23].

**Figure 1:**
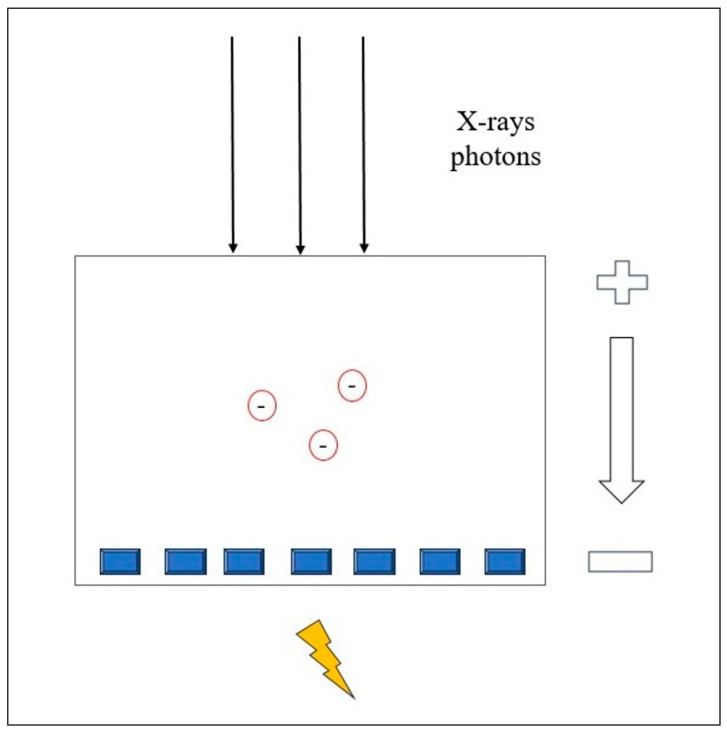
Physical principle of the Photon-counting Detector CT.

Each X-ray photon is promptly converted into electron-hole pairs, which move toward the anode under an applied voltage (Figure 1). The charge carriers collected by the pixels generate a second electrical signal proportional to the incoming X-ray photons.

Thus, the signal from the PCDs carries the energy information about each individually detected photon [24,25].

Many advantages arise from the PCCT energy-discriminating ability. By setting the energy thresholds, the PCCT can separate photons that exceed a certain level, thereby reducing the electronic noise. This can be excluded from the count data by choosing the low-energy threshold to be slightly higher than the energy level associated with the electronic noise signal amplitude. However, electronic noise can have a positive effect on the detected energy spectrum because its signal amplitude is added to that of the detected photon, which consequently increases its energy. This can be beneficial for examinations with low detector signal intensities, such as those performed with a low radiation dose [24,25].

Because each individual photon is sorted according to its energy level at the PCD CT, an energy bin image can be reconstructed using only higher energy photons. Compared with conventional EID CT images, high-energy-resolution images are more immune to beam-hardening effects in areas around dense bones and calcium. Moreover, the combination of using the high-energy bin image and tin beam filtration can reduce metal artifacts, providing improved delineation for tissue regions [22].

The spatial resolution obtainable with any conventional CT detector is mainly determined by the detector element size, with smaller ones improving spatial resolution. Because PCDs do not have scintillators, they can be fabricated with smaller elements than EIDs [23]. PCD CT system has an effective detector pixel size of 0.25 mm × 0.25 mm with a spatial resolution to be limited to 150 μm [22].

Another main driving force of PCD CT is that it allows the acquisition of simultaneous multienergy (>2) CT images. User-defined energy threshold selection provides the freedom to select the correct energy thresholds tailored to a specific diagnostic task. This unique feature enables single-source, single-tube potential, single-acquisition, single-detector layer, and single-filter multi-energy CT imaging with perfect temporal and spatial registration in the acquired multi-energy data, eliminating many artifacts [22]. By selecting energy thresholds lower and higher than the K edge of a specific contrast agent, PCD CT may enable K-edge imaging [22].

Due to these latter considerations, PCCT allows simultaneous assessment of different CM, both iodinated and non-iodinated such as gadolinium and bismuth [26]. In addition to the published research, new experimental findings with a CM that incorporates tungsten are included [27]. However, only a few animal studies or experimental phantoms on bi-contrast imaging and new CM are currently available, owing to ethical precautions. For example, some of these studies have focused on models for studying the heart, liver, and bowel [28,29,30].

## 3. Photon-Counting CT—Endoleaks Detection

Abdominal aortic aneurysm (AAA) is a bulging of the abdominal aorta with a diameter ≥ 3 cm, affecting 1.6–7.2% of people and occurring in 0.4–0.7% per year in the Western population [31]. The current criteria for elective treatment of AAAs are based on the aortic diameter. The European Society for Vascular Surgery (ESVS) guidelines suggest elective repair for AAAs ≥ 5.5 cm in men (5 cm in women) or if they show rapid growth (greater than 1 cm/year). Elective repair is also recommended for asymptomatic fusiform AAAs measuring 5.5 cm in men and 5.0 cm in women [32]. Endovascular aneurysm repair (EVAR) is a common treatment that involves the insertion of a covered stent graft inside the aneurysm [33]. However, EVAR can lead to endoleaks (ELs), which are the persistence of blood flow outside the graft into the aneurysm sac, posing the risk of growth and rupture (Supplementary Table S1). ELs was the most common complication (53% of all complications), with an incidence of 11.7% [34,35]. Traditional CT protocols include unenhanced, arterial-phase, and venous/delayed-phase scans to assess the blood vessels and stent grafts [36,37,38]. However, repeated CT scans expose patients to high radiation doses and kidney toxicity, necessitating the exploration of advanced imaging techniques to mitigate this concern [39].

Photon-counting CT (PCCT) has emerged as a promising advanced imaging modality aimed at reducing radiation exposure with comparable while improving endoleak detection accuracy. Turrion Gomollon et al. [10] conducted a comparative study to evaluate the image quality and endoleak detection on PCCT in 110 patients after EVAR. This study compared traditional triphasic CT (comprising the true non-contrast, arterial, and venous phases) with a novel approach using virtual non-iodine (VNI) images in a biphasic CT protocol. Two radiologists independently assessed the presence of endoleaks, and the findings were compared with a reference standard. The results indicate that both imaging protocols were equally effective in detecting endoleaks, demonstrating a high sensitivity and specificity (Supplementary Table S2). The inter-observer agreement was substantial, further validating the results. In addition, the image noise levels of the two protocols were comparable, with the VNI images showing slightly lower noise levels. This study suggests that using VNI images in a biphasic CT protocol can provide equivalent endoleak detection and image quality, similar to the traditional triphasic CT approach. This study demonstrated the feasibility and potential of using virtual non-iodine image PCCT as a valuable tool for reducing radiation exposure during endoleak detection.

Cosset et al. [12] has explored the potential of bicolor K-edge PCCT in endoleak analysis by employing a dynamic thoracic aorta phantom. They created three common thoracic endoleak types using iodinated and gadolinium contrast agents, and aimed to assess the feasibility of identifying and characterizing these endoleaks. The imaging protocol involved a two-phase contrast agent injection with iodinated contrast, followed by gadolinium injection. Spectral imaging successfully differentiated the distribution of these contrast agents, revealing early and late blood flow patterns for different endoleak types. Bicolor K-edge imaging and SPCCT allowed the characterization of endoleaks within the thoracic aorta in a single acquisition combined with a biphasic contrast agent injection. The authors underscored the potential of SPCCT to accurately characterize endoleaks and offer valuable insights for improved diagnosis and management.

**Figure 2:**
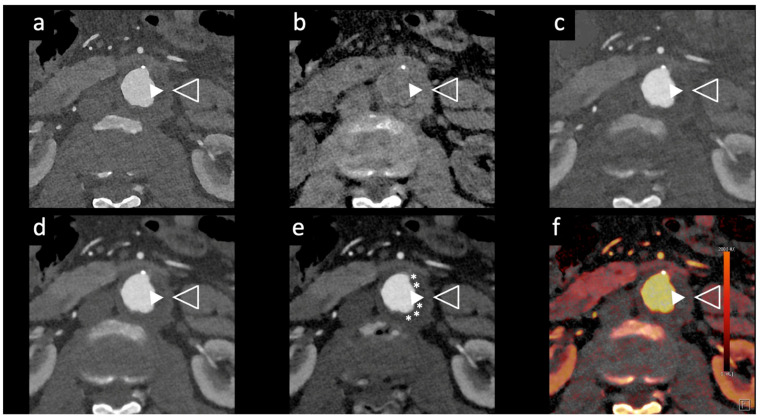
Comparison of image quality of an abdominal aortic aneurysm evaluated with photon-counting CT and standard contrast media (axial images). A thickened aortic wall related to aortitis (between solid and empty arrowhead) and intraluminal thrombotic stratification (* white asterisks) are visible at the level of the abdominal aortic aneurysm. High Resolution evaluation (Matrix 1024 × 1024) (**a**); Virtual Non-Contrast VCN (**b**); Iodine Map (**c**); 55 keV reconstruction (**d**); Pure Lumen reconstruction (**e**); Spectral Dual Energy Reconstruction (**f**). Department of Radiology, Fondazione Toscana Gabriele Monasterio, Pisa, Italy.

**Figure 3:**
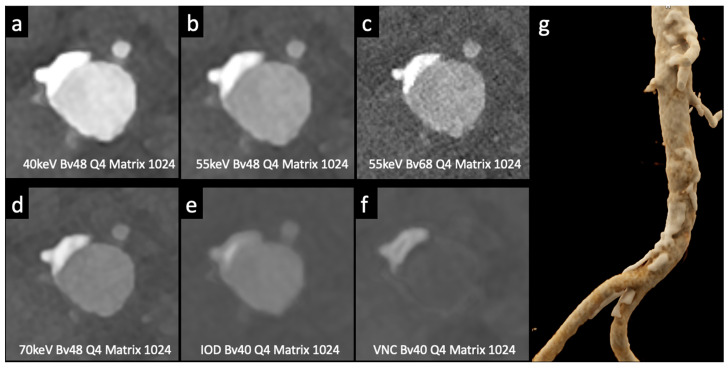
Comparison of image quality with photon-counting CT and standard contrast media. Abdominal aortic lumen with wall calcification axial images. High Resolution images (Matrix 1024 × 1024) with different reconstruction kernels (window level W2000, C700): 40 keV Kernel Bv48, Q4 (**a**); 55 keV Kernel Bv48, Q4 (**b**); 55 keV Kernel Bv68, Q4 (**c**); 70 keV Kernel Bv48, Q4 (**d**); Iodine map reconstruction, kernel Bv40, Q4 (**e**); Virtual Non-Contrast VCN reconstruction (**f**); 3D-Cinematic Rendering of the abdominal aorta (**g**). Department of Radiology, Fondazione Toscana Gabriele Monasterio, Pisa, Italy.

Dangelmaier et al. [11] explored the feasibility of PCCT with two contrast agents to detect endoleaks following EVAR. Using a specialized abdominal aortic aneurysm (AAA) phantom filled with a mixture of iodine, gadolinium, and calcium chloride, they were able to differentiate the distribution of these agents, enabling the reliable detection of endoleaks. Using an SPCCT prototype scanner with multi-energy bins, the results of this study showed that SPCCT has the potential to replace multiphase CT scans for endoleak detection without sacrificing diagnostic accuracy. It distinguishes endoleaks from calcifications in a single scan, thereby significantly reducing radiation exposure, which is vital in clinical settings (Figure 2 and Figure 3).

Overall, these studies highlight ongoing advancements in endoleak detection in patients with AAAs undergoing EVAR. PCCT holds promise for improving Els management by reducing radiation exposure while maintaining accuracy.

## 4. Photon-Counting CT Aortic Imaging: Radiation Dose and Contrast Volume Reduction

PCCT has shown significant promise, particularly in patients with chronic kidney disease (CKD) or those requiring follow-up imaging. Several recent studies have demonstrated the potential benefits of PCCT, including excellent image quality, reduced contrast agent volume, and improved contrast-to-noise ratio (CNR).

**Figure 4:**
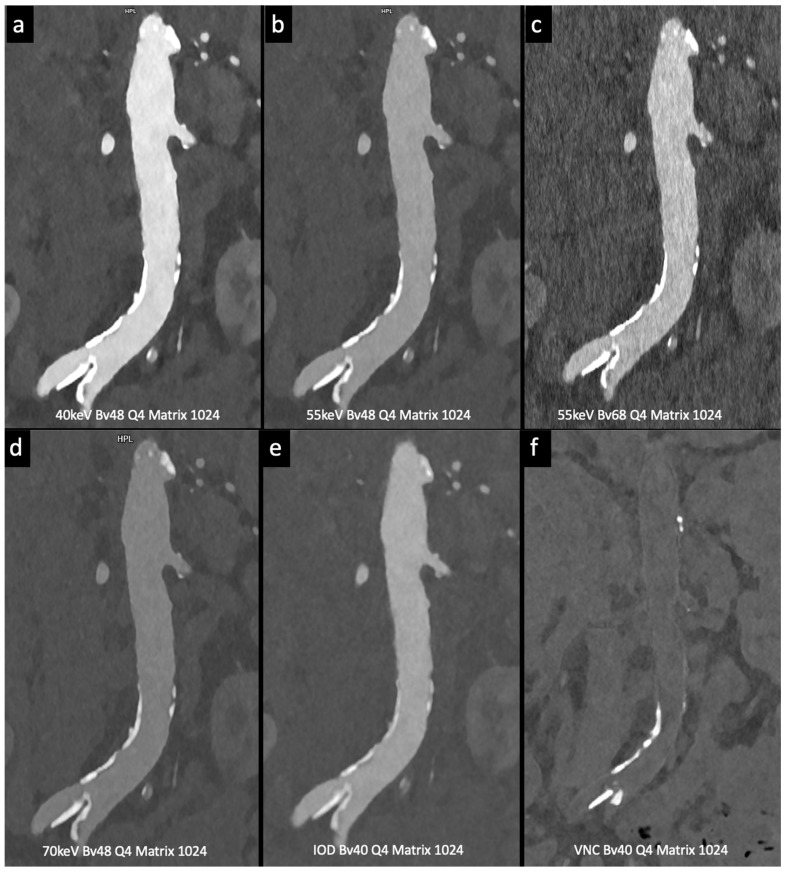
Comparison of image quality with photon-counting CT and standard contrast media. Abdominal aortic lumen with wall calcification coronal images. High Resolution images (Matrix 1024 × 1024) with different reconstruction kernels (window level W2000, C700): 40 keV Kernel Bv48, Q4 (**a**); 55 keV Kernel Bv48, Q4 (**b**); 55 keV Kernel Bv68, Q4 (**c**); 70 keV Kernel Bv48, Q4 (**d**); Iodine map reconstruction, kernel Bv40, Q4 (**e**); Virtual Non Contrast VCN reconstruction (**f**). Department of Radiology, Fondazione Toscana Gabriele Monasterio, Pisa, Italy.

Higashigaito et al. [13] explored a low-volume contrast medium protocol for thoracoabdominal CT angiography using PCCT. This study compared PCCT with previous energy-integrating detector (EID) CT at equal radiation doses. Virtual monoenergetic images (VMI) at 50 keV exhibited the best trade-off between objective and subjective image quality, with a 25% higher CNR than that of EID CT. The low-volume contrast media protocol also reduced the volume of the contrast medium by 25%. These findings suggest that PCCT with a low-volume contrast media protocol achieves superior CNR while maintaining non-inferior image quality compared to EID CT (Figure 4).

Decker et al. [18] evaluated the potential of virtual non-contrast reconstructions using a calcium-preserving algorithm (VNCPC) compared with the standard algorithm (VNCConv) in patients after EVAR. This study demonstrated that VNCPC reconstructions had excellent image quality with complete contrast removal and minimal stent and calcification removal errors. Compared with VNCConv, VNCPC showed higher image noise, but significantly better subjective image quality. Aortic contrast removal was complete in all the VNCPC reconstructions. Readers considered VNCPC suitable for replacing true non-contrast scans in 95% of the cases, whereas VNCConv was suitable in only 75% of the cases. In conclusion, this study demonstrated that VNCPC reconstructions exhibit excellent image quality with complete contrast removal and minimal erroneous subtraction, making them a potential alternative to true non-contrast acquisitions.

Euler et al. [19] used virtual monoenergetic images (VMI) at 40 keV and 45 keV in photon-counting CT (PCCT) and compared them to traditional CT with matched radiation doses. PCCT with VMI showed a significant improvement in contrast-to-noise ratio (CNR), especially in overweight patients. The subjective image quality ratings varied slightly, with some differences in the vessel attributes and noise. Overall, high-pitch PCCT with VMI at 40 keV and 45 keV provided a substantial CNR advantage over conventional CT at equivalent radiation doses, benefiting overweight patients. This study suggests that VMI at 45–50 keV offers a favorable balance between objective and subjective image quality.

MRI, including 4D flow MRI [40], offers significant benefits in aortic imaging, such as multiplanar imaging without ionizing radiation or iodine CM, making it an alternative to CT scans, in a non-emergency setting [41]. In fact, challenges persist with MRI due to long scan times, the metallic-related artifacts, the lower spatial resolution and the impossibility of calcium assessment, complicating its widespread clinical use in particular in the acute setting [42,43].

**Figure 5:**
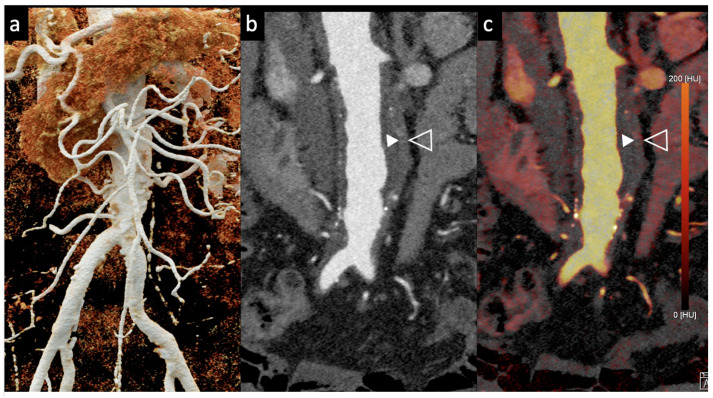
Aortic angiographic evaluation of the abdominal aortic aneurysm evaluated with photon-counting CT and standard contrast media (the same example of Figure 2, coronal view). The aortic wall is indicated between solid and empty arrowhead. 3D-Volume Rendering representation (**a**); High-Resolution coronal evaluation, 55 keV, Kernel Bv68 Q4 Matrix 1024 (**b**); Spectral Dual Energy coronal Reconstruction (**c**). Department of Radiology, Fondazione Toscana Gabriele Monasterio, Pisa, Italy.

Rau et al. [14] presented a case study of an 81-year-old patient with incipient chronic renal failure who required contrast-enhanced aortoiliac CT angiography for follow-up imaging of an asymptomatic abdominal aortic aneurysm (AAA). They employed a first-generation PCCT scanner to reduce contrast agent use while maintaining diagnostic reliability. Utilizing a PCCT protocol with dual-source spectral image acquisition and dynamic monochromatic reconstruction near the K-edge of iodine, this study demonstrated a significantly reduced required contrast agent while preserving diagnostic confidence. This study shows promise for minimizing renal damage during imaging. However, further research is needed to refine these protocols and post-processing techniques. A similar case is shown in Figure 2 and Figure 5.

Niehoff et al. [15] conducted a study on the first clinically approved PCCT scanner, assessing the diagnostic reliability of Virtual Non Contrast (VNC) images compared to TNC images. This retrospective study included 72 patients and analyzed the consistency and quantitative properties of VNC images reconstructed from arterial and portal venous phases against TNC images. The results showed that the mean difference in Hounsfield units (HU) between the VNC and TNC images was less than 4 HU across all tissues (aorta included), except spongious bone. Based on these findings the algorithm for iodine subtraction fundamentally works, although it requires refinement for accurate clinical application, and caution is recommended when using VNC images in routine practice.

Emrich et al. [16] conducted a study to test the reduction in iodinated CM volumes in coronary CT angiography using a first-generation dual-source PCCT system with a dynamic circulation phantom. By progressively reducing the concentration of the CM in a 50 mL bolus, they examined the impact on image quality. They found that the diagnostic image quality could be maintained with a 50% reduction in CM concentration, achieving sufficient attenuation at energy levels between 40 and 55 keV. The best CNR was observed at 40 keV for all CM concentrations. However, reducing CM concentration to 20% of the initial concentration resulted in an inadequate attenuation.

A study by Cundari et al. [17] aimed to determine the optimal energy level for VMIs and assess the possibility of reducing CM in coronary computed tomography angiography (CCTA) using PCCT. Group 1 was scanned using a standard CM protocol, which served as the basis for determining the best VMI energy level. VMIs from 40 to 60 keV were analyzed for objective image quality (IQ) subjectively by two blind readers. The best VMI level for IQ was 45 keV. Groups 2 and 3 had 20% and 40% reductions in CM volume, respectively. Group 2 showed no IQ difference from the standard, whereas Group 3 had a slightly lower IQ but remained diagnostic. The study concluded that PCCT can allow for significant CM reduction without compromising the diagnostic IQ on CCTA.

In summary, PCCT technology, particularly beneficial for patients with CDK or those requiring follow-up imaging, has been shown to provide high-quality images with reduced CM volumes and improved CNR. The studies by Higashigaito et al. [13], Rau et al. [14], Niehoff et al. [15], and Emrich et al. [16] converge on the finding that PCCT with optimized protocols significantly lowers CM usage without sacrificing image quality. In particular, the application of a low-volume CM protocol and VMI techniques, such as 40–45 keV reconstructions, ensures diagnostic reliability, which could reduce the risk of renal damage and aid in safer long-term patient monitoring.

## 5. Limitations

PCCT offers superior spatial and contrast resolution compared with conventional CT, but its aortic imaging applications are limited. Its high sensitivity to artifacts, especially from metal prostheses, and longer scan times due to its reliance on patient physiology present challenges [1,44]. The scarcity of suitable cases for rare diseases, the lack of specialized software, and absence of standardized processing protocols also hinder its wider clinical use. In addition, the high cost of PCCT systems, necessitating multiple generators and X-ray tubes, limits their affordability and adoption [1,23,45]. Moreover, the lack of molecular imaging agents for clinical applications does not permit an advance tissue characterization. Research to refine PCCT protocols and post-processing techniques is essential to improve the diagnostic capabilities of aortic imaging.

## 6. Conclusions

In conclusion, PCCT holds significant promise in the field of aortic imaging and offers numerous advantages over conventional CT techniques. This review demonstrates the potential of PCCT in aortic imaging. PCCT has higher spatial and contrast resolutions than conventional CT, allowing endoleak detection with less radiation after EVAR. PCCT can also characterize endoleaks in the thoracic aorta with bicolor K-edge imaging and dual-contrast agents and reduce contrast agent volumes with low-volume protocols, benefiting patients with CKD or those needing frequent CT imaging. Furthermore, PCCT can improve CNR with VIM at optimal energy levels, thereby achieving higher image quality and lower contrast-related risks. These advancements in imaging techniques hold promise for optimizing patient care and diagnosis in the management of aortic pathologies. Further research and extensive studies are needed to confirm these results and explore the full potential of PCCT in aortic imaging.

## Figures and Tables

**Table 1:** Study selected from our research.

Endoleaks detection	Turrion Gomollon et al. Investigative Radiology 2023 [10]	Retrospective study (110 patients)	PCCT Endoleak detection and image quality were comparable to biphasic CT. Reduction of scan phases and radiation exposure.
	Cosset et al. Diagnostic and Interventional Imaging 2023 [12]	Phantom experimental study	PCCT allows characterization of thoracic endoleaks (I-III) in a single acquisition with a biphasic contrast agent (gadolinium+iodine)
	Dangelmaier et al. European Radiology 2018 [11]	Phantom experimental study	PCCT may replace multiphase CT to capture endoleak dynamics. PCCT allow distinction from intra-aneurysmatic calcifications. Reduction of radiation exposure.
Contrast Media Volume Reduction	Higashigaito et al. Radiology Cardiothoracic Imaging 2023 [13]	Prospective study (100 patients)	PCCT was associated with higher CNR, low-volume contrast media protocol. Noninferior image quality compared with EID CT at the same radiation dose.
	Rau et al. Radiology Case Reports 2023 [14]	Case report (follow-up imaging of AAA)	PCCT modified scan protocol allowed a significant reduction of contrast agent while preserving diagnostic confidence
	Niehoff et al. Diagnostics 2021 [15]	Retrospective study (72 patients)	The use of VNC images, versus TNC images, requires refinement for accurate clinical application, and caution it is recommended in routine practice.
	Emrich et al. Investigative Radiology 2023 [16]	Phantom experimental study	Diagnostic image quality in coronary and aorta PCCT angiography can be maintained with a 50% reduction in CM concentration.
	Cundari et al. Academic Radiology 2023 [17]	Retrospective study (100 patients)	45 keV setting for VMI in coronary PCCT is optimal, and PCCT enables up to 40% CM reduction while maintaining diagnostic image quality.
Radiation Dose Reduction	Decker et al. Diagnostics 2022 [18]	Retrospective study (20 patients) after EVAR	PCCT have high image quality and should reduce cumulative radiation dose in patients post-EVAR.
	Euler et al. Investigative Radiology 2022 [19]	Prospective study (40 patients)	High-pitch PCCT with VMI at 40 and 45 keV resulted in increased CNR compared with EID-CT with ATVS at matched radiation dose. CNR gain of PCCT increased in overweight patients.

Abbreviations: TNC = True Non-contrast; AAA = abdominal aortic aneurysm; EID = Energy-Integrating Detector; CNR = Contrast to Noise Ratio; ATVS = Automatic Tube Voltage Selection, VNC = virtual non-contrast, VMI = Virtual monoenergetic images.

## Data Availability

Data sharing is not applicable to this article.

## Supplementary Materials

The following supporting information can be downloaded at: https://www.mdpi.com/article/10.3390/tomography10010001/s1, Table S1: Endoleak classification [37,38,39,46]; Table S2: Sensitivity and Specificity of EL detection comparison [10].

## Abbreviations

AAA	abdominal aortic aneurysm
CAD	coronary artery disease
CNR	contrast-to-noise ratio
CTA	computed tomography angiography
EID	energy-integrating detector
ELs	Endoleaks
EVAR	Endovascular Aortic Repair
PCCT	Photon-Counting CT
PCD	Photon-Counting Detector
TNC	True Non-Contrast
VMI	Virtual monoenergetic images
VNI	Virtual Non-Iodine image

## References

[1] Meloni A., Frijia F., Panetta D., Degiorgi G., De Gori C., Maffei E., Clemente A., Positano V., Cademartiri F. (2023). Photon-Counting Computed Tomography (PCCT): Technical Background and Cardio-Vascular Applications. Diagnostics.

[2] Rubin G.D., Leipsic J., Schoepf U.J., Fleischmann D., Napel S. (2014). CT Angiography after 20 Years: A Transformation in Cardiovascular Disease Characterization Continues to Advance. Radiology.

[3] Pepe A., Crimì F., Vernuccio F., Cabrelle G., Lupi A., Zanon C., Gambato S., Perazzolo A., Quaia E. (2023). Medical Radiology: Current Progress. Diagnostics.

[4] Manghat N., Morgan-Hughes G., Roobottom C. (2005). Multi-detector row computed tomography: Imaging in acute aortic syndrome. Clin. Radiol..

[5] Schlett C.L., Pursnani A., Marcus R.P., Truong Q.A., Hoffmann U. (2014). The Use of Coronary CT Angiography for the Evaluation of Chest Pain. Cardiol. Rev..

[6] Yoon Y.E., Wann S. (2011). Evaluation of Acute Chest Pain in the Emergency Department. Cardiol. Rev..

[7] Meloni A., Cademartiri F., Pistoia L., Degiorgi G., Clemente A., De Gori C., Positano V., Celi S., Berti S., Emdin M. (2023). Dual-Source Photon-Counting Computed Tomography—Part III: Clinical Overview of Vascular Applications beyond Cardiac and Neuro Imaging. J. Clin. Med..

[8] Sun Z. (2012). Cardiac CT imaging in coronary artery disease: Current status and future directions. Quant. Imaging Med. Surg..

[9] Rajagopal J.R., Farhadi F., Richards T., Nikpanah M., Sahbaee P., Shanbhag S.M., Bandettini W.P., Saboury B., Malayeri A.A., Pritchard W.F. (2021). Evaluation of Coronary Plaques and Stents with Conventional and Photon-counting CT: Benefits of High-Resolution Photon-counting CT. Radiol. Cardiothorac. Imaging.

[10] Gomollon A.M.T., Mergen V., Sartoretti T.M., Polacin M.M., Nakhostin D., Puippe G., Alkadhi H., Euler A. (2023). Photon-Counting Detector CT Angiography for Endoleak Detection After Endovascular Aortic Repair. Investig. Radiol..

[11] Dangelmaier J., Bar-Ness D., Daerr H., Muenzel D., Si-Mohamed S., Ehn S., Fingerle A.A., Kimm M.A., Kopp F.K., Boussel L. (2018). Experimental feasibility of spectral photon-counting computed tomography with two contrast agents for the detection of endoleaks following endovascular aortic repair. Eur. Radiol..

[12] Cosset B., Sigovan M., Boccalini S., Farhat F., Douek P., Boussel L., Si-Mohamed S.A. (2023). Bicolor K-edge spectral photon-counting CT imaging for the diagnosis of thoracic endoleaks: A dynamic phantom study. Diagn. Interv. Imaging.

[13] Higashigaito K., Euler A., Eberhard M., Flohr T.G., Schmidt B., Alkadhi H. (2022). Contrast-Enhanced Abdominal CT with Clinical Photon-Counting Detector CT: Assessment of Image Quality and Comparison with Energy-Integrating Detector CT. Acad. Radiol..

[14] Rau S., Soschynski M., Schlett C.L., Hagar M.T. (2023). Spectral aortoiliac photon-counting CT angiography with minimal quantity of contrast agent. Radiol. Case Rep..

[15] Niehoff J.H., Woeltjen M.M., Laukamp K.R., Borggrefe J., Kroeger J.R. (2021). Virtual Non-Contrast versus True Non-Contrast Computed Tomography: Initial Experiences with a Photon Counting Scanner Approved for Clinical Use. Diagnostics.

[16] Emrich T., O’Doherty J., Schoepf U.J., Suranyi P., Aquino G., Kloeckner R., Halfmann M.C., Allmendinger T., Schmidt B., Flohr T. (2023). Reduced Iodinated Contrast Media Administration in Coronary CT Angiography on a Clinical Photon-Counting Detector CT System. Investig. Radiol..

[17] Cundari G., Deilmann P., Mergen V., Ciric K., Eberhard M., Jungblut L., Alkadhi H., Higashigaito K. (2023). Saving Contrast Media in Coronary CT Angiography with Photon-Counting Detector CT. Acad. Radiol..

[18] Decker J.A., Bette S., Scheurig-Muenkler C., Jehs B., Risch F., Woźnicki P., Braun F.M., Haerting M., Wollny C., Kroencke T.J. (2022). Virtual Non-Contrast Reconstructions of Photon-Counting Detector CT Angiography Datasets as Substitutes for True Non-Contrast Acquisitions in Patients after EVAR—Performance of a Novel Calcium-Preserving Reconstruction Algorithm. Diagnostics.

[19] Euler A., Higashigaito K., Mergen V., Sartoretti T.B., Zanini B., Schmidt B., Flohr T.G., Ulzheimer S., Eberhard M.M., Alkadhi H.M. (2022). High-Pitch Photon-Counting Detector Computed Tomography Angiography of the Aorta. Investig. Radiol..

[20] Verdun F., Racine D., Ott J., Tapiovaara M., Toroi P., Bochud F., Veldkamp W., Schegerer A., Bouwman R., Giron I.H. (2015). Image quality in CT: From physical measurements to model observers. Phys. Medica.

[21] Stein T., Rau A., Russe M.F., Arnold P., Faby S., Ulzheimer S., Weis M., Froelich M.F., Overhoff D., Horger M. (2023). Photon-Counting Computed Tomography—Basic Principles, Potenzial Benefits, and Initial Clinical Experience. RöFo—Fortschritte Auf Dem Geb. Röntgenstrahlen Bildgeb. Verfahr..

[22] Willemink M.J., Persson M., Pourmorteza A., Pelc N.J., Fleischmann D. (2018). Photon-counting CT: Technical Principles and Clinical Prospects. Radiology.

[23] Tortora M., Gemini L., D’Iglio I., Ugga L., Spadarella G., Cuocolo R. (2022). Spectral Photon-Counting Computed Tomography: A Review on Technical Principles and Clinical Applications. J. Imaging.

[24] Leng S., Bruesewitz M., Tao S., Rajendran K., Halaweish A.F., Campeau N.G., Fletcher J.G., McCollough C.H. (2019). Photon-counting Detector CT: System Design and Clinical Applications of an Emerging Technology. RadioGraphics.

[25] Rajendran K., Petersilka M., Henning A., Shanblatt E.R., Schmidt B., Flohr T.G., Ferrero A., Baffour F., Diehn F.E., Yu L. (2022). First Clinical Photon-counting Detector CT System: Technical Evaluation. Radiology.

[26] Tao S., Rajendran K., McCollough C.H., Leng S. (2019). Feasibility of multi-contrast imaging on dual-source photon counting detector (PCD) CT: An initial phantom study. Med. Phys..

[27] Jost G., McDermott M., Gutjahr R., Nowak T., Schmidt B., Pietsch H. (2023). New Contrast Media for K-Edge Imaging With Photon-Counting Detector CT. Investig. Radiol..

[28] Symons R., Cork T.E., Lakshmanan M.N., Evers R., Davies-Venn C., Rice K.A., Thomas M.L., Liu C.-Y., Kappler S., Ulzheimer S. (2017). Dual-contrast agent photon-counting computed tomography of the heart: Initial experience. Int. J. Cardiovasc. Imaging.

[29] Muenzel D., Daerr H., Proksa R., Fingerle A.A., Kopp F.K., Douek P., Herzen J., Pfeiffer F., Rummeny E.J., Noël P.B. (2017). Simultaneous dual-contrast multi-phase liver imaging using spectral photon-counting computed tomography: A proof-of-concept study. Eur. Radiol. Exp..

[30] Ren L., Rajendran K., Fletcher J.G., McCollough C.H., Yu L. (2020). Simultaneous Dual-Contrast Imaging of Small Bowel With Iodine and Bismuth Using Photon-Counting-Detector Computed Tomography. Investig. Radiol..

[31] Aggarwal S., Qamar A., Sharma V., Sharma A. (2011). Abdominal Aortic Aneurysm: A Comprehensive Review. Exp. Clin. Cardiol..

[32] Wanhainen A., Verzini F., Van Herzeele I., Allaire E., Bown M., Cohnert T., Dick F., van Herwaarden J., Karkos C., Koelemay M. (2019). Editor’s Choice—European Society for Vascular Surgery (ESVS) 2019 Clinical Practice Guidelines on the Management of Abdominal Aorto-iliac Artery Aneurysms. Eur. J. Vasc. Endovasc. Surg..

[33] Keisler B., Carter C. (2015). Abdominal Aortic Aneurysm. Am. Fam. Physician.

[34] Gozzo C., Caruana G., Cannella R., Farina A., Giambelluca D., Dinoto E., Vernuccio F., Basile A., Midiri M. (2022). CT angiography for the assessment of EVAR complications: A pictorial review. Insights Imaging.

[35] Isselbacher E.M., Preventza O., Black J.H., Augoustides J.G., Beck A.W., Bolen M.A., Braverman A.C., Bray B.E., Brown-Zimmerman M.M., Chen E.P. (2022). 2022 ACC/AHA Guideline for the Diagnosis and Management of Aortic Disease: A Report of the American Heart Association/American College of Cardiology Joint Committee on Clinical Practice Guidelines. Circulation.

[36] Hong C., Heiken J.P., Sicard G.A., Pilgram T.K., Bae K.T. (2008). Clinical Significance of Endoleak Detected on Follow-Up CT After Endovascular Repair of Abdominal Aortic Aneurysm. Am. J. Roentgenol..

[37] Lehmkuhl L., Andres C., Lücke C., Hoffmann J., Foldyna B., Grothoff M., Nitzsche S., Schmidt A., Ulrich M., Scheinert D. (2013). Dynamic CT Angiography after Abdominal Aortic Endovascular Aneurysm Repair: Influence of Enhancement Patterns and Optimal Bolus Timing on Endoleak Detection. Radiology.

[38] Reginelli A., Capasso R., Ciccone V., Croce M.R., Di Grezia G., Carbone M., Maggialetti N., Barile A., Fonio P., Scialpi M. (2016). Usefulness of triphasic CT aortic angiography in acute and surveillance: Our experience in the assessment of acute aortic dissection and endoleak. Int. J. Surg..

[39] Partovi S., Trischman T., Rafailidis V., Ganguli S., Rengier F., Goerne H., Rajiah P., Staub D., Patel I.J., Oliveira G. (2018). Multimodality imaging assessment of endoleaks post-endovascular aortic repair. Br. J. Radiol..

[40] Takehara Y., Sekine T., Obata T. (2022). Why 4D Flow MRI? Real Advantages. Magn. Reson. Med. Sci..

[41] Riambau V., Böckler D., Brunkwall J., Cao P., Chiesa R., Coppi G., Czerny M., Fraedrich G., Haulon S., Jacobs M. (2017). Editor’s Choice—Management of Descending Thoracic Aorta Diseases. Eur. J. Vasc. Endovasc. Surg..

[42] Takahashi K., Sekine T., Ando T., Ishii Y., Kumita S. (2022). Utility of 4D Flow MRI in Thoracic Aortic Diseases: A Literature Review of Clinical Applications and Current Evidence. Magn. Reson. Med. Sci..

[43] Colacchio E.C., Berton M., Squizzato F., Menegolo M., Piazza M., Grego F., Antonello M. (2023). The role of multimodal imaging in emergency vascular conditions: The journey from diagnosis to hybrid operating rooms. Semin. Vasc. Surg..

[44] Cademartiri F., Meloni A., Pistoia L., Degiorgi G., Clemente A., De Gori C., Positano V., Celi S., Berti S., Emdin M. (2023). Dual-Source Photon-Counting Computed Tomography—Part I: Clinical Overview of Cardiac CT and Coronary CT Angiography Applications. J. Clin. Med..

[45] Alizadeh L.S., Vogl T.J., Waldeck S.S., Overhoff D., D’angelo T., Martin S.S., Yel I., Gruenewald L.D., Koch V., Fulisch F. (2023). Dual-Energy CT in Cardiothoracic Imaging: Current Developments. Diagnostics.

[46] Kazimierczak W., Serafin Z., Kazimierczak N., Ratajczak P., Leszczyński W., Bryl Ł., Lemanowicz A. (2019). Contemporary imaging methods for the follow-up after endovascular abdominal aneurysm repair: A review. Videosurgery Other Miniinvasive Tech..

