# Supplementary material for: Advantages of Photon-Counting Detector CT in Aortic Imaging
Source: Tomography. 2023 Dec 19;10(1):1–13. doi: 10.3390/tomography10010001 (PMC10821336; doi:10.3390/tomography10010001)
Supplement: Supplementary file 1 [file tomography-10-00001-s001.zip › tomography-2698805-supplementary.pdf]

**Supplementary Table S1.** Endoleak classification.

| | | |
|---|---|---|
| ***Type 1 Endoleak (EL1)*** | 5-10% | **EL1a**: leakage from proximal attachment site of the graft[37] |
| | | **EL1b**: leakage from distal attachment site of the graft |
| | | **EL1c**: leakage from back filling due to an incomplete common iliac arterial occlusion |
| ***Type 2 Endoleak (EL2)*** | 10-40% | Backflow of collateral arteries into the aneurysm sac[38] |
| ***Type 3 Endoleak (EL3)*** | 2-4% | Stent graft component separation or EL due to a fabric tear[39] |
| ***Type 4 Endoleak (EL4)*** | Almost never seen with new generation grafts | Leakage due to porosity of the graft[46] |
| ***Type 5 Endoleak (EL5),*** *also known as* ***"endotension"*** | Diagnosis of exclusion | Expansion of the sac without an apparent EL on imaging[46] |

**Supplementary Table S2.** Sensitivity and Specificity of EL detection comparison

| **Study** | Sensitivity | Specificity |
|---|---|---|
| **Turrion Gomollon** et al. *Investigative Radiology* 2023[10] | Reader 1: TNC 0.95 vs VNI 0.95<br>Reader 2: TNC 0.88 vs VNI 0.88 | Reader 1: TNC 0.84 vs VNI 0.86<br>Reader 2: TNC 0.98 vs VNI 0.94 |

**Abbreviations:** TNC = True Non-contrast; VNI = Virtual Non-iodine;
